# Oxidative Stress, Mutations and Chromosomal Aberrations Induced by In Vitro and In Vivo Exposure to Furan

**DOI:** 10.3390/ijms22189687

**Published:** 2021-09-07

**Authors:** Maria Teresa Russo, Gabriele De Luca, Nieves Palma, Paola Leopardi, Paolo Degan, Serena Cinelli, Gaetano Pepe, Pasquale Mosesso, Emma Di Carlo, Carlo Sorrentino, Piero Musiani, Riccardo Crebelli, Margherita Bignami, Eugenia Dogliotti

**Affiliations:** 1 National Centre for Chemical Products, Cosmetics and Consumer Protection, Istituto Superiore di Sanità, Viale Regina Elena 299, 00161 Rome, Italy; russo@iss.it; 2 Oncology and Molecular Medicine, Istituto Superiore di Sanità, Viale Regina Elena 299, 00161 Rome, Italy; deluca@iss.it; 3 Department of Environment and Health, Istituto Superiore di Sanità, Viale Regina Elena 299, 00161 Rome, Italy; nievespalma@yahoo.es (N.P.); Paola.leopardi15.pl@gmail.com (P.L.); riccardo.crebelli@gmail.com (R.C.); 4 IRCCS AOU San Martino, Istituto Nazionale per la Ricerca sul Cancro, 16132 Genoa, Italy; paolo.degan@hsanmartino.it; 5 European Research Biology Center, Via Tito Speri 12/14, Pomezia, 00071 Rome, Italy; s.cinelli@erbc-group.com; 6 Dipartimento di Scienze Ecologiche e Biologiche, Università degli Studi della Tuscia, 01100 Viterbo, Italy; pepe@unitus.it (G.P.); mosesso@unitus.it (P.M.); 7 Department of Medicine and Sciences of Aging, “G. d’Annunzio” University of Chieti-Pescara, 66022 Chieti, Italy; emma.dicarlo@unich.it (E.D.C.); carlo.sorrentino@unich.it (C.S.); 8 Anatomic Pathology and Immuno-Oncology Unit, Center for Advanced Studies and Technology (CAST), “G. d’Annunzio” University of Chieti-Pescara, 66022 Chieti, Italy

**Keywords:** furan, mutagenicity, clastogenicity, oxidative DNA base damage, inter-strand cross-links

## Abstract

Furan is a volatile compound that is formed in foods during thermal processing. It is classified as a possible human carcinogen by international authorities based on sufficient evidence of carcinogenicity from studies in experimental animals. Although a vast number of studies both in vitro and in vivo have been performed to investigate furan genotoxicity, the results are inconsistent, and its carcinogenic mode of action remains to be clarified. Here, we address the mutagenic and clastogenic activity of furan and its prime reactive metabolite cis-2 butene-1,4-dial (BDA) in mammalian cells in culture and in mouse animal models in a search for DNA lesions responsible of these effects. To this aim, Fanconi anemia-derived human cell lines defective in the repair of DNA inter-strand crosslinks (ICLs) and *Ogg1*^−/−^ mice defective in the removal of 8-hydroxyguanine from DNA, were used. We show that both furan and BDA present a weak (if any) mutagenic activity but are clear inducers of clastogenic damage. ICLs are strongly indicated as key lesions for chromosomal damage whereas oxidized base lesions are unlikely to play a critical role.

## 1. Introduction

Furan is a volatile organic chemical that is formed during the processing or cooking of many common foods. In addition, it is found in tobacco smoke and is used as a synthetic intermediate in the production of pesticides, stabilizers, and solvent for resins and pharmaceuticals. The widespread human exposure to furan through the diet is a public health concern because of its hepatotoxicity and carcinogenicity in rats and mice [1,2,3,4]. Oral exposure to furan caused tumors at several different tissue sites in mice and rats. A two-year administration of furan by gavage to F-344 rats and B6C3F1 mice produced a dose-dependent increase in hepatocellular adenoma and carcinomas in mice and rats of both sexes. In addition, cholangiocarcinoma and pheochromocytoma were also observed in both sexes of rats and mice, respectively [2]. In a more recent National Center for Toxicological Research study where a lower dose range of furan was used, only cholangiofibrosis was observed with no evidence of neoplastic findings [5,6]. Most of the cholangiocarcinomas in rats reported in the National Toxicology Program (NTP) study [2] were later reclassified as cholangiofibrosis and cholangiocarcinomas were confirmed only at the highest furan dose [5]. Furan has been classified as a possible human carcinogen (Group 2B) by the International Agency for Research on Cancer (IARC) [3] and “reasonably anticipated to be a human carcinogen” by the NTP [1].

The mechanism of furan-induced liver tumorigenicity is unclear, with uncertainties on the relative involvement of genotoxic and non-genotoxic components. The currently available data on the genotoxicity of furan are contradictory. Furan did not induce gene mutations in bacteria [7] with the exception of a weak positive effect in *Salmonella typhimurium* strain TA100 [8]. Both positive and negative results were obtained in the L5178Y *Tk*^+^/*Tk*^−^ mouse lymphoma forward mutation assay [9,10] and in Chinese hamster ovary and V79 cells for induction of chromosomal damage [2,11,12]. A similarly complex picture on furan genotoxicity can be drawn when gene and chromosomal mutations as well as other genetic endpoints (e.g., DNA single strand breaks) were investigated in vitro or in vivo [2,12,13,14,15,16,17,18,19].

The current hypothesis for the mechanism of furan-induced carcinogenesis is the metabolic activation of furan, mostly via hepatic cytochrome P450 2E1, to a highly reactive and cytotoxic intermediate, i.e., cis-2 butene-1,4-dial (BDA) [20,21]. This can readily react with thiol and aminogroups of glutathione, with amino acids as well as with DNA in vitro [22,23,24,25]. Studies on the genotoxicity of BDA provided more consistent positive results. These include the ability of BDA to form dC and dA adducts in DNA isolated from calf thymus and BDA-treated bacteria [26], DNA single-strand breaks (SSBs) [10,27] and DNA-protein cross-links [27] in mammalian cells. BDA induces direct mutations in *Salmonella typhimurium* TA104 strain [26,28,29] and in L5178Y *Tk*^+^/*Tk*^−^ cells (without metabolic activation) [10].

The possibility that some of the genotoxic effects of furan are secondary to oxidative stress induced by furan administration has been also proposed. Indeed, increased levels of oxidatively damaged DNA persist in areas of cholangiofibrosis in rats long after furan exposure has been interrupted [30] and the chronic exposure to furan generates in DNA oxidized purines and pyrimidines as detected by the enzyme-modified comet assay [31]. Thus, it is important to define whether furan genotoxic properties depend on the ability of this compound to generate oxidatively damaged DNA or the result of its direct or metabolite-driven interaction with DNA.

From this brief overview it emerges that, although the genotoxicity of furan and BDA has been widely addressed in bacteria and mammalian cells in vitro and in vivo, the mechanism of induction of genotoxic effects is far from being clarified. This study is designed to gain insights into the lesions underlying the genotoxic effects of furan and BDA with a special focus on oxidatively induced DNA damage and DNA inter-strand cross links (ICLs).

## 2. Results

### 2.1. Furan Is Not Mutagenic at the Hprt and Tk Loci and Is a Strong Inducer of DNA Oxidation in Mammalian Cell Lines

**Figure 1:**
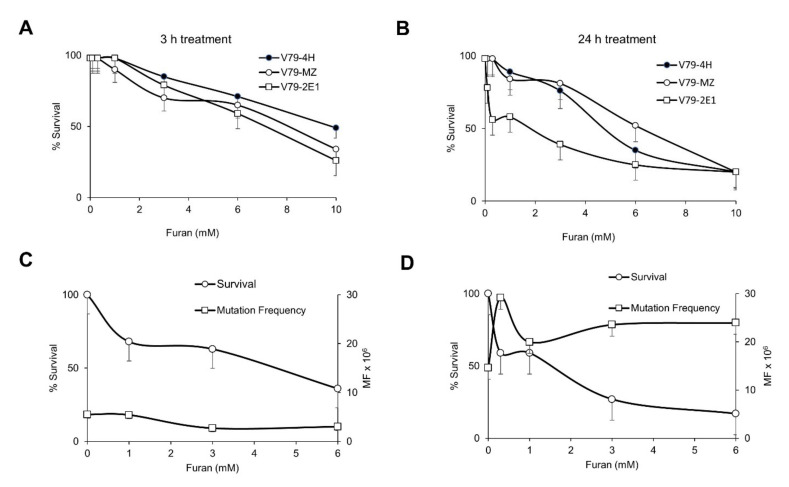
Cytotoxicity and *Hprt* mutation frequency induced by furan in a panel of V79 cell lines, namely V79-4H, V79-MZ and V79 as detailed in the legend. (**A**) Cytotoxicity after 3 h and (**B**) 24 h exposure at the indicated doses; (**C**,**D**) cytotoxicity and *Hprt* mutation frequency after 24 h exposure at the indicated doses in V79-4H (**C**) and V79-2E1 (**D**) cell lines. Furan surviving cells are expressed as percentage of the surviving untreated cells. Mutation frequencies (MF) are calculated as described in M&M. Bars indicate SD.

To evaluate the mutagenic potential of furan in mammalian cells we selected the V79-4H cell line and an isogenic V79 cell system formed by the genetically engineered V79MZh2E1 cell line expressing human CYP2E1 (indicated as V79-2E1 from now on) and its parental V79MZ counterpart. This model system was chosen because of the evidence that furan CYP450 2E1-catalyzed oxidation to BDA is involved in furan-induced toxicity both in vitro and in vivo [reviewed in 4]. After 2 h exposure to furan, the three V79 derived cell lines showed similar toxicities, with a marked decrease in cell survival at high doses (>6 mM) (Figure 1A). Following a 24 h exposure to furan, V79-2E1 cells showed some hypersensitivity in comparison to the parental V79MZ and V79-4H cells in the low range of furan doses (0.1–0.3 mM) (Figure 1B). This suggests that the capacity of biotransformation of furan within this CYP2E1-expressing cell line, although limited, leads to the formation of a toxic metabolite. We note that these experimental conditions might cause a reduction of oxygen which is required for CYP-catalyzed metabolic activation of furan.

Mutation induction by furan at the *Hprt* gene was then evaluated. No increase in mutation frequency was detected in V79-4H cells treated with a wide range of furan doses for either 24 (Figure 1C) or 3 h (data not shown). After 24 h exposure to furan a large variation in the mutagenic response of V79-2E1 cells was observed in five independent experiments. An indication of a slight increase in mutation frequency was found only at the lowest dose (0.3 mM) (the same where an enhancement in killing had been detected) (Figure 1D). In view of the inefficient metabolic capacity of these cells and their extremely high and variable spontaneous mutation frequency (11.6 ± 5.3 × 10^−6^), these should be considered overall negative results.

Mutation induction at the *Tk* locus was also investigated in the L5178Y mouse lymphoma cell line. In this cell line furan was reported to be mutagenic in the absence of metabolic activation [9] but these results were not confirmed in a successive study that showed lack of mutagenic activity both in the absence as well as in the presence of metabolic activation [10]. Here, cells were exposed to furan for 24 h in the absence of metabolic activation. In two independent experiments a dramatic increase in cytotoxicity was observed in a very narrow range of doses (around 5 mM). Since increases in mutation frequency were detected at highly cytotoxic doses, i.e., 3–11% of Relative Total Growth (RTG), these results can be considered negative (OECD Test Guideline N. 490). The results are presented in Table 1.

**Figure 2:**
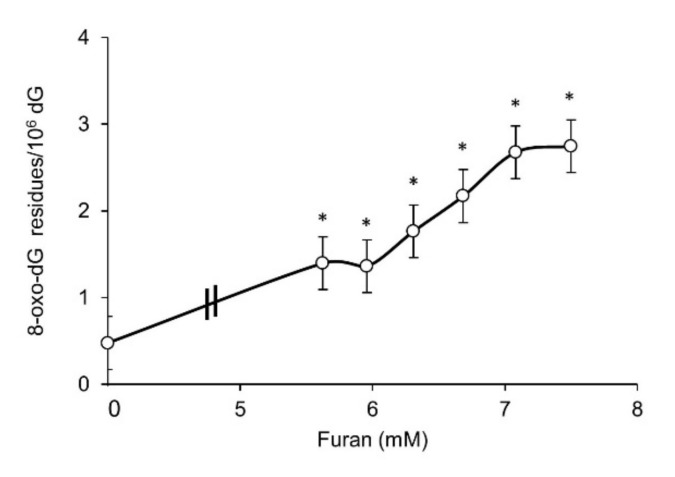
DNA 8-oxodG levels in L5178Y cells after 24 h exposure to furan at the indicated doses as measured by HPLC/EC. DNA 8-oxodG levels are expressed × 10^6^ dG. Bars indicate SD. * *p* < 0.05 by Student *t*-test.

Oxidative stress induced DNA damage can result in toxicity and genomic instability. Since guanine is the most susceptible base to oxidation, the formation of DNA 8-hydroxyguanine (8-oxodG) was measured by HPLC/EC in L5178Y cells following 24 h exposure to furan (Figure 2). A marked dose-related increase in DNA 8-oxodG levels is observed above 5.5 mM, the same dose where a drastic decrease in survival occurs. An approximately six-fold increase in DNA 8-oxodG levels is detected at the highest dose tested (7.5 mM) when compared to untreated cells.

Overall, these data indicate that furan *per se* exerts a high cytotoxic effect associated with a significant and dose-related increase in DNA guanine oxidation. In the absence of metabolic activation, furan is not mutagenic at two gene loci, namely at the *Hprt* and *Tk* genes in V79 and L5178Y cells, respectively.

### 2.2. The Furan Metabolite BDA Is a Weak Mutagen at the Hprt and Tk Loci and a Poor Inducer of DNA Oxidation in Mammalian Cell Lines

**Figure 3:**
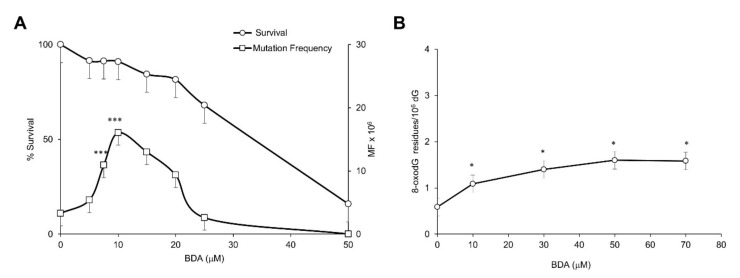
Cytotoxicity, *Hprt* mutation frequency and DNA base oxidation induced by BDA in V79-4H cell line. (**A**) Cytotoxicity and *Hprt* mutation frequency after 3 h exposure to BDA at the indicated doses; (**B**) DNA 8-oxodG levels after 3 h exposure to BDA at the indicated doses as measured by HPLC/EC. Furan surviving cells are expressed as percentage of the surviving untreated cells. Mutation frequencies (MF) are calculated as described in M&M. DNA 8-oxodG levels are expressed × 10^6^ dG. Bars indicate SD. * *p* < 0.05; *** *p* < 0.0001 by Student *t*-test.

The cytotoxic and mutagenic effects of the active form of furan, BDA, were investigated in V79-4H cell line. A significant (*p* ≤ 0.0001) dose-related increase in mutation frequency was observed in the absence of cytotoxicity after 3 h exposure to a narrow range of doses (up to 10 μM). Above this dose the decrease in survival paralleled that observed in mutation frequency (Figure 3A).

The induction of oxidative DNA damage was explored in a wide dose range (up to 70 μM). BDA exposure induced a doubling of 8-oxodG levels at a dose associated with <10% toxicity (10 μM) but no further increase of DNA oxidation was observed at higher cytotoxic doses (Figure 3B).

In the *Tk* mutation assay in L5178Y cells, BDA induced large increases in mutant frequencies at concentrations from 50 µM onwards where very high levels of cytotoxicity were observed with a RTG below or equal to 10%. Since at these levels of cytotoxicity mechanisms other than direct DNA damage can lead to positive results, they were excluded from further evaluations including statistical analyses. At lower concentrations, dose-related increases in mutation frequencies, compared with the negative control, were observed. At 40 μM (experiment 1) the observed increase reached a statistical significance. However, the induced mutation frequency did not exceed the Global Evaluation Factor (see M&M) and the observed increase was not reproduced in the second experiment. In both experiments a highly significant dose-related increase of the small colonies’ mutation frequency was observed which reflects a potential clastogenic effect. The results are presented in Table 2.

In conclusion these results provide indication of a mild mutagenic effect in a narrow range of doses at the *Hprt* locus in V79 cells whereas data in the mouse lymphoma *Tk* mutation assay are inconclusive due to high cytotoxicity. BDA is a poor inducer of oxidative DNA damage even at high cytotoxic doses.

### 2.3. BDA Induces Micronuclei in L5178Y Lymphoblastoid Cells

The potential clastogenic effects of BDA were tested by performing the micronucleus assay in the L5178Y cell line. Dose-related and statistically significant increases in the frequencies of micronucleated cells were observed following treatment with BDA for either 4 or 24 h (Table 3). The micronuclei were characterized by a small dimension in line with the occurrence of clastogenic events. At the selected dose-levels the observed cytotoxicity was low in the short (4 h) treatment (10–27% of the concurrent negative control) and moderate after the 24 h treatment (49–62%). These levels of cytotoxicity do not exceed the level of 50% ± 5 that is indicated by the OECD Test Guideline N. 487 to avoid false positive outcome. The positive control Mitomycin-C (MMC) at 1.5 µg/mL induced statistically significant increase in micronucleated cells indicating the correct functioning of the test system.

These data indicate that BDA induces a clastogenic effect in L5178Y cells as suggested by the significant increase of small *Tk* mutant colonies.

### 2.4. Furan Induces Chromosomal Aberrations in the Presence of Both S9 Metabolism and a Defect in DNA ICL Repair

The above described data raise the following questions (i) what is the role of CYP1E1 metabolism in the induction of clastogenic effects, and (ii) what are the DNA lesions involved? To answer these questions, we investigated the ability of furan to induce chromosomal aberrations in cell lines defective in the repair of DNA ICLs both in the absence and in the presence of S9 fractions.

To this aim lymphoblastoid cell lines derived from Fanconi anemia (FA) patients which bear defects in the *FANCA* gene (LFA55^−/−^ and LFA145^−/−^) and their normal counterpart, LFA 195^++^ cell line, derived from a healthy relative of one FA patient (LFA145^−/−^) were used (see M&M). FA cells have inactivating mutations in a signaling pathway that protects genomic integrity from the DNA damage caused by cross-linking agents [32].

**Figure 4:**
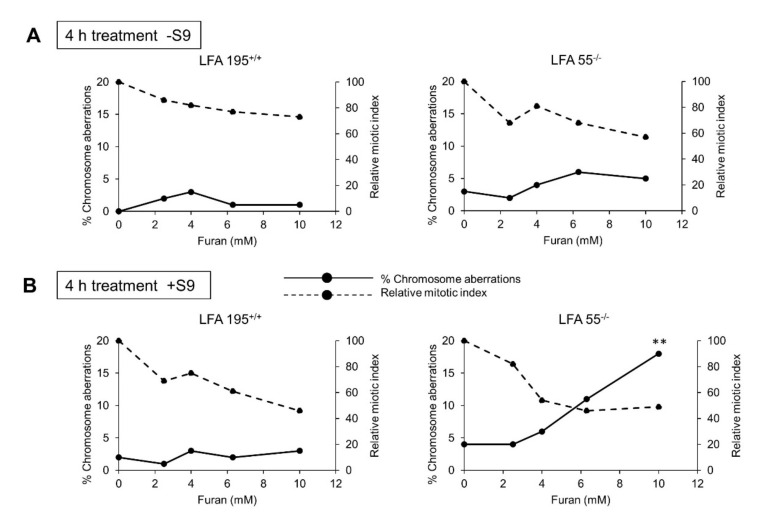
Chromosomal aberrations induced by 4 h exposure to furan in immortalized lymphoblastoid cell lines originated from a “normal” individual (LFA195^+/+^) or a patient with FA (LFA55^−/−^). (**A**) Treatments performed in the absence of S9 metabolic activation. (**B**) Treatments performed in the presence of S9 metabolic activation. Rat livers S9 tissue homogenates, prepared from animals induced with isoniazid to accumulate high levels of cytochrome P450 2-E1. ** *p* < 0.01.

Following a 4 h exposure to furan a marked dose-related increase in the number of aberration-bearing cells was observed in the LFA55^−/−^ cell line in the presence of S9 metabolism (Figure 4). No effects were observed in the wild-type lymphoblastoid cell line LFA 195^++^ cell line

The observed increase reached statistical significance (*p* < 0.01) at the highest dose-level assayed (10 mM). The induced cytotoxicity evaluated by the reduction of mitotic indices compared to the negative controls showed an ideal reduction at the highest dose-level (10 mM) of approximately 50% (OECD Test Guideline N. 473) (Figure 4A).

No induction of chromosomal aberrations was observed in either LFA55^−/−^ or the wild-type LFA195^+/+^ cell lines in the absence of S9 metabolism (Figure 4B). These data indicate that furan is not directly reactive to DNA but induces chromosomal damage possibly through the metabolic conversion to the clastogenic intermediate BDA [21].

### 2.5. A Defect in the Repair of DNA ICLs Significantly Increases the Frequency of Chromosomal Aberrations Induced by BDA

**Figure 5:**
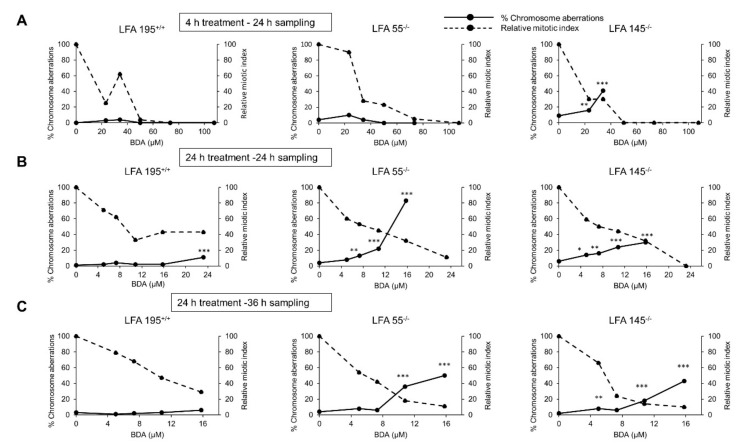
Chromosomal aberrations induced in the absence of S9 metabolism by BDA in immortalized lymphoblastoid cell lines originated from a normal individual (LFA195^+/+^) or from patients with FA (LFA55^−/−^ or LFA145^−/−^) with different mutations in the *FANCA* gene (see M&M). (**A**) Treatments performed for 4 h and sampling of cells at 24 h from the beginning of treatment; (**B**) Treatments performed for 24 h and sampling of cells at 24 h from the beginning of treatment; (**C**) Treatments performed for 24 h with a recovery period of 12 h and sampling of cells at 36 h from the beginning of treatment in order to assess DNA repair in normal and FA lymphoblastoid cell lines. * *p* < 0.05; ** *p* < 0.01; *** *p* < 0.001.

Following 4 h treatment with BDA (24 h sampling), statistically significant increases in chromosomal damage were only observed in one of the two FA cell lines employed, i.e., LFA145^−/−^ (Figure 5A). When BDA was administered for 24 h (24 h sampling) (Figure 5B), marked dose-related and statistically significant increases in chromosomal damage were observed in both FA cell lines (LFA55^−/−^ and LFA145^−/−^) and also in the normal LFA195^+/+^ cell line but only at the highest non-toxic dose tested (23.2 μM). In order to corroborate these findings and evaluate the efficiency of DNA ICLs repair, following 24 h treatment, an additional 12 h recovery was allowed (36 h sampling) (Figure 5C). No increase in the frequency of aberration-bearing cells was observed in the normal LFA195^+/+^ cells indicating that repair of BDA-induced DNA damage has occurred thus preventing further chromosomal damage. In contrast, statistically significant levels of aberration-bearing cells were observed in the LFA55^−/−^ and LFA145^−/−^ FA cells thus confirming that FA cells are defective in the repair of BDA-induced DNA lesions. These data provide strong evidence that BDA induced clastogenic lesions are ICL.

### 2.6. Subacute Oral Administration of Furan to C57BL6 Mouse Strain Causes Liver Toxicity

To investigate whether a sub-acute oral administration of furan induced any toxicity in the C57BL6 mouse strain, three furan doses, including those tested in the NTP long-term bioassay (8 and 15 mg/kg bw), were administered by gavage to groups of 4-month-old C57BL6 mice (5–6 animals/group), 5 days a week for 28 days. No signs of overt toxicity related to furan administration were recorded during the treatment period. The death of one animal from the 8 mg/kg bw group was considered unrelated to the furan treatment. In comparison to mice receiving the vehicle alone, a slight increase in relative liver weights was recorded in mice at all furan doses. The increases in the hepatic index reached statistical significance at 8 and 15 mg furan/kg bw/day (*p* < 0.05 and *p* < 0.001, respectively) (Table 4). In contrast no alteration in the splenic index was observed at sacrifice.

**Figure 6:**
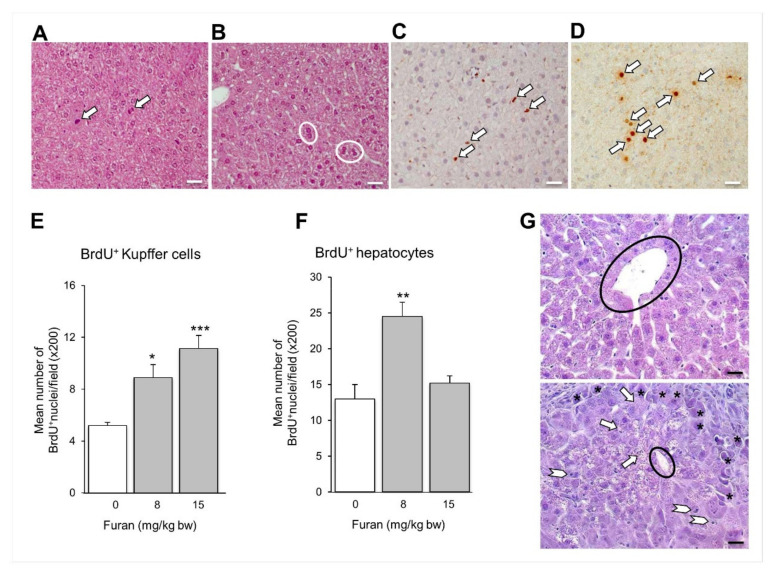
Immunopathological features of liver damage associated with furan administration. H&E staining shows the presence of mitoses (arrows in (**A**)) and binucleated hepatocytes (circles in (**B**)) after the administration of low-dose furan. Intranuclear accumulation of BrdU highlights the increased proliferative activity of Kupffer cells (arrows in (**C**), data in (**E**)) and hepatocytes (arrows in (**D**), data in (**F**)). (**G**) H&E staining shows the signs of severe liver damage which follows the administration of high-dose furan (**bottom**) compared to normal tissue (**top**): fatty degeneration (arrows), apoptotic figures (arrowheads) and frequent necrotic hepatocytes at the periphery of the lobule (asterisks). Black circles: central veins. * *p* = 0.0140, ** *p* = 0.0056, *** *p* = 0.0008, Student’s *t*-test vs. 0. Magnification: (**A**–**D**), ×400; (**G**), ×630. Scale bars (**A**–**D**): 20 μm; (**G**): 10 μ m.

The hepatic toxicity was evaluated by histopathological examination of liver samples from furan-treated and control mice. At low doses (8–15 mg/kg bw), we found only signs of mild liver damage (periportal vacuolation), along with evidence of regenerative response, such as mitoses and frequent binucleated hepatocytes (Figure 6A,B). Furthermore, there was an increase in the proliferative activity of both Kupffer cells and hepatocytes, as demonstrated by the intranuclear accumulation of 5-bromo-2-deoxyuridine (BrdU) (Figure 6C–F). However, at the highest dose (30 mg/kg bw) the hepatic lobules showed evident signs of severe damage, such as fatty degeneration (marked by the deposition of fat globules in the hepatocytes), frequent apoptotic figures and zonal necrosis at the periphery (Figure 6G).

In conclusion a 28-day sub-acute oral furan administration induced a dose-dependent increase in liver toxicity, with increased cell proliferation occurring at low doses and necrosis at the highest dose. In the same experimental conditions, no signs of toxicity were recorded in the spleen.

### 2.7. Subchronic Exposure to Furan Results in a Moderate Increase in Micronucleus Frequency in Splenocytes with No Signs of Spleen Toxicity

The ability of furan to induce micronuclei was investigated in cytokinesis-blocked spleen lymphocytes of mice treated with the same range of furan doses used in the subchronic oral toxicity experiment (8–30 mg/kg/bw) (Table 5). A statistically significant increase (ranging from 1.45 up to 3.3-fold) in the frequency of micronucleated cells, was observed in the spleen of furan-treated mice in two independent experiments. No influence of treatment on target cell viability and ability to proliferate was indicated by the lack of variations in the nuclear division index (NDI) (data not shown). Thus, similarly to the results obtained in B6C3F1 mice [15] a subchronic exposure to furan results in a moderate increase in micronucleus frequency with no signs of spleen toxicity also in C57BL6 mice.

### 2.8. Furan-Induced Chromosomal Damage in the Spleen Is Independent from Oxidatively Induced DNA Damage

**Figure 7:**
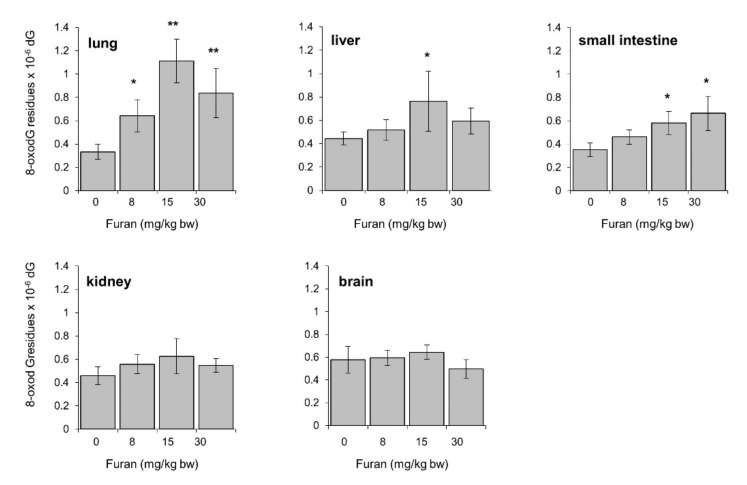
Levels of 8-oxodG in lung, liver, small intestine, kidney, brain DNA in C57Bl6 mice following a 28-day oral exposure to increasing furan concentrations. The amount of 8-oxodG residues/10^6^ dG was measured in genomic DNA by HPLC/EC (see Section 4). All values are mean +/− SE (5–11 animals/group). Asterisks indicate significant differences (* *p* < 0.05; ** *p* < 0.01 by two-tailed Student’s *t*-test).

To study whether furan treatment was associated with induction of oxidative damage to DNA in vivo, levels of DNA 8-oxodG were analyzed by HPLC-EC in several organs of C57BL6 mice following a 4-week oral exposure to furan (dose range: 8–30 mg/kg/bw). Consistently with previous results in this mouse strain steady-state levels of the oxidized purine ranged from 0.3 up to 0.57 8-oxodG residues × 10^−6^ dG in different organs (Figure 7) [33]. Dose-dependent increases in DNA 8-oxodG were observed in the lung and small intestine of furan-treated mice (3.4-fold and 1.7-fold over background levels, respectively) (Figure 7). In contrast no dose-response was observed in the liver, where a significant 1.7-fold increase in DNA 8-oxodG was limited to the 15 mg/Kg bw dose where the highest effect on hepatic index (liver weight/body weight %) was recorded (Table 4). No evidence of 8-oxodG induction was reported in kidney and brain.

**Figure 8:**
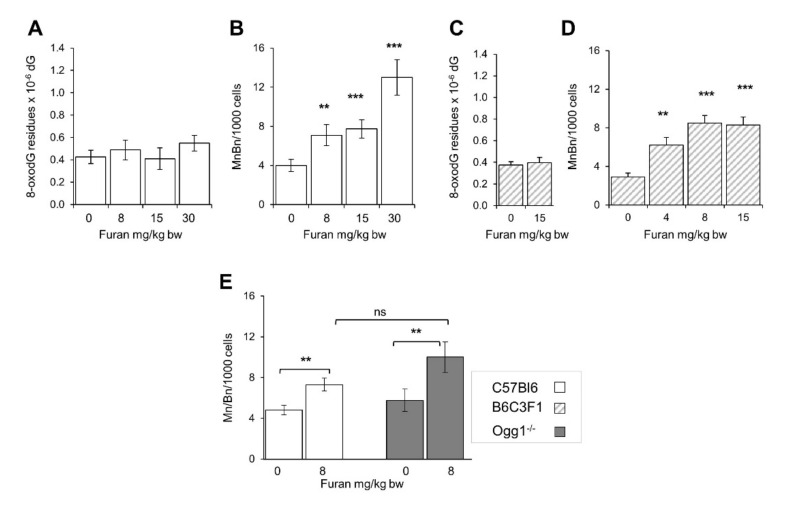
Induction of micronucleated binucleated cells and DNA 8-oxodG in the spleen of C57Bl6 and B6C3F1 following a 28-day oral exposure to furan. Frequency of Bin/Min/1000 splenocytes induced in C57Bl6 mice following exposure to the indicated furan doses (**A**); Levels of 8-oxodG/residues/10^6^ dG in spleen DNA from furan-treated C57Bl6 (**B**) and B6C3F1 mice (**C**); Frequency of MnBn/1000 splenocytes in furan treated C57Bl6 mice (hatched bars) are taken from Leopardi et al., 2010 (**D**); Frequency of MnBn/1000 splenocytes in furan treated C57Bl6 mice (open bars) and *Ogg1*^−/−^ mice (closed bars) (**E**). All values are mean +/− SE (5–11 animals/group). Asterisks indicate significant differences (** *p* < 0.01, *** *p* < 0.001 by two-tailed Student’s *t*-test), ns, not significant; MnBn, Micronucleated binucleatedcells.

Since furan increased the frequency of micronuclei in splenocytes, levels of DNA 8-oxodG were also investigated in the spleen of furan treated C57BL6 mice. No increase of the oxidized purine level was observed in this organ at furan doses which result in significant increases in micronuclei frequency (Figure 8A,B).

An increase in the frequency of binucleate splenocytes has also been previously reported in another mouse strain, the B6C3F1 mice [15]. An analysis of DNA 8-oxodG levels in the spleen of furan-treated B6C3F1 confirmed that also in this strain of mice furan does not induce measurable changes in DNA oxidation levels (Figure 8C,D).

Finally, we compared the levels of micronuclei in wild-type and *Ogg1*^−/−^ *C57BL6* mice exposed to 8 mg/kg/bw furan. A similar increase in the micronuclei frequency was observed in the two genotypes indicating that the clastogenic DNA lesions induced by furan are not the substrate for this repair enzyme (Figure 8E).

## 3. Discussion

Although several studies have addressed the genotoxicity of furan, the results are controversial, and the underlying mechanisms need to be clarified. Here, by using in vitro and in vivo models, we provide clear evidence that furan, via conversion to BDA, exerts a clastogenic activity most likely through formation of ICLs. In addition, our data indicate that oxidative DNA damage is unlikely to be a critical step in this process.

At first the mutational activity of furan and BDA was investigated in mammalian cell lines. In the absence of metabolic activation furan was not mutagenic neither at the *Hprt* gene in hamster V79 cells nor at the *Tk* gene in mouse lymphoma L5178Y cells. Mutation analysis was challenged by the dramatic drop in survival occurring in a very narrow range of doses and increases in mutation frequency were detected only at unacceptably low RTG (<10%). This problem together with conditions of cell exposure in closed systems (due to the high volatility of furan) may underlie the controversial data reported in the literature using the same mutation assays [9,10].

A lack of mutational activity was also reported in vivo in *gpt* delta rats treated with furan at carcinogenic doses [19]. No changes in the frequency of *gpt* (point mutations) and *Spi*^−^ (small deletions) mutants were observed in the liver, the target organ for carcinogenesis. Similarly, furan did not increase mutation frequency at the liver *cII* gene upon exposure of female B6C3F1 Big Blue transgenic mice to furan, although changes in the mutation spectrum were reported, namely GC>TA transversions [17].

Notwithstanding the technical problems inherent to mammalian cell studies, overall, the evidence of mutational activity by furan both in vitro and in vivo is weak (if any).

The analysis of the mutational activity of BDA, a prime reactive metabolic intermediate of furan, was also challenged by high cytotoxicity. This is not surprising considering that BDA readily reacts with thiol and amino groups of vital cell components. A weak but dose-related increase of mutation frequency was observed in the *Hprt* assay (but only in a narrow range of doses), while mutation data in the *Tk* assay were inconclusive due to the rapid drop in cell survival. It is of note that a dose-related increase in the percentage of mutant *Tk* small colonies was consistently observed indicating a potential clastogenic effect. In line with this observation, we show that BDA induces micronuclei in L5178Y cells.

Previous studies reported mutagenic activity of BDA in bacteria [29] and in the mouse lymphoma *Tk* assay at non-toxic doses [10]. In contrast, a lack of increase in mutation frequency was observed at the *cII* locus of Big Blue mouse embryo fibroblasts even though a concentration-dependent shift in mutational spectrum (increase in AT>CG transversions) was reported [17].

Overall, these data indicate that both furan and BDA are at best weak mutagens both in vitro and in vivo and the analysis of mutational spectra does not allow to identify the mutagenic DNA lesions.

More consistent data were obtained when the clastogenic activity of furan and BDA was explored. Here, we show that a 4-week oral exposure to furan is associated with a dose-related increase in micronuclei frequency in splenocytes of C57Bl6 mice. These results are in agreement with the in vitro clastogenic properties of furan active metabolite revealed by BDA-induced micronuclei in L5178Y cells. They also confirm previous in vivo observations in splenocytes of B6C3F1 mice and F344 rats chronically exposed to furan [15,34]. Interestingly, these positive results in the spleen are paralleled by consistently negative results in bone marrow and peripheral blood micronucleus assays [16,31,34].

In line with furan-induced free radical production [35] (here, we show that furan induces a marked dose-related DNA guanine oxidation (approximately a 6-fold increase over endogenous levels of DNA 8-oxodG) when mammalian cells are exposed in vitro. In our cells this phenomenon seems to be independent of conversion of furan to BDA as this last one is a poor inducer of oxidative DNA damage. In addition, the extensive analysis of DNA 8-oxodG levels in several organs of furan-treated mice is indicative of some oxidative stress affecting the lung, liver, and small intestine. The ability of furan to induce oxidized purines in the liver is not surprising since increased levels of *Fpg*-sensitive sites have been shown in the comet assay following a relatively short exposure (4 days) of F344 rats to furan doses used in cancer bioassay [31]. Here, we show that this DNA damage is more extensive and is not restricted to an acute treatment but persists after a chronic exposure to furan.

Histopathological data in the liver suggest that the oxidative stress may play a key role in the onset of mild hepatic damage which follows the administration of low-dose furan, while it is not involved in the pathogenesis of massive necrosis that occurs after administration of high-doses. Thus, at low furan doses (8–15 mg/kg bw), there was evidence of a regenerative response, i.e., mitoses, binucleated hepatocytes, and enhanced hepatocytes and Kupffer cells proliferation, associated with a significant increase of DNA 8-oxodG levels. These data agree with the reported co-localization of immunostaining for DNA 8-oxo-dG and the Ki67 marker of cell proliferation in the liver of 3-day furan-treated rats [30]. However, at the highest dose (30 mg/kg bw), where necrosis prevailed, no increase in DNA 8-oxodG levels could be identified.

The open question remains: which are the lesions responsible for furan clastogenicity? Our data clearly demonstrate that furan-induced chromosomal damage in the spleen is independent from oxidatively induced DNA damage. Thus, no increase in the levels of DNA 8-oxodG, a well-established marker of oxidative damage to DNA, was identified in the spleen of either C57Bl6 or B6C3F1 mice, at doses where clear increases in micronuclei frequency were observed. In addition, no significant differences in micronuclei frequency were found in splenocytes of furan treated wild-type and *Ogg1*^−/−^ mice, a mouse strain defective in the removal of 8-oxodG from DNA.

The molecular mechanism underlying furan/BDA-induced chromosomal damage has been proposed to be due to the formation of ICLs. These lesions, if left unrepaired, can lead to mutations, chromosome breakage and mitotic catastrophe [32]. This hypothesis is supported by in vitro studies with BDA and oligonucleotides [36] and by alkaline elution data in CHO cells [27]. More importantly, the induction of ICLs by furan was probed by a modified comet assay in which splenocytes from chronically exposed B6C3F1 mice were irradiated in vitro with γ-rays before electrophoresis [15]. In addition, foci of phosphorylated histone H2AX (γ-H2AX), which might represent intermediates in the repair of DNA ICLs, were identified in splenocytes as well as in liver cells. An increase in DNA ICLs could also be identified in the liver of B6C3F1 mice following an acute treatment with an extremely high furan dose (250 mg/kg/bw) [14].

Here, we provide further support to these observations by data in FA cells which are defective in the repair of ICLs. By using this model cell system, we show that the induction of chromosomal aberrations by furan is strictly dependent on S9 microsomal fraction and is amplified by the defect in ICLs repair. This allows us to conclude that BDA is the reactive clastogenic intermediate of furan and ICLs are likely to be the key lesion in this event.

In conclusion, our findings support a model where the ability of furan to induce gene mutations is, at the most, a minor contributor to the carcinogenicity of furan in the liver. We show here that furan/BDA induce chromosomal damage both in vitro and in vivo and we propose that ICLs, but not oxidative DNA damage, are the lesions involved in furan clastogenicity. Despite the final demonstration of furan genotoxicity in the target organ for hepatocarcinogenesis is still lacking, overall, these results provide valuable information for the elucidation of the furan mode of action and risk characterization.

## 4. Materials and Methods

### 4.1. Chemicals

Furan (C4H4O, CAS 110-00-9) was purchased from Sigma-Aldrich Italia (Milan, Italy). The product tested (cat. no. 18,592-2) was >99% pure, stabilized with 0.025% of butylated hydroxytoluene. The BDA furan metabolite was obtained from its precursor 2,5-diacetoxy-2,5-dihydrofuran (kindly provided by Dr. W. Dekant, University of Würzburg, Germany) by hydrolysis in water for 24 h at room temperature [28].

### 4.2. Preparation of In Vitro Metabolic Activation System (S9)

Rat livers S9 tissue homogenates, prepared from young male rats following induction with isoniazid to accumulate high levels of cytochrome P450 2E1, the major enzyme involved in furan biotransformation, were obtained from Biopredic International (Rennes, France).

### 4.3. Cell Lines

A set of V79 Chinese hamster lung fibroblasts cell lines was selected for this study: the V79-4H cell line, extensively used for mutation assays, and an isogenic V79 cell system constituted by a genetically engineered cell line expressing human CYP2E1, named V79MZh2E1 and its parental cell line, V79MZ (GenPharmTox Bio Tech AG, Martinsried, Germany). V79 cells were grown in Eagle minimal essential medium supplemented with 2 mM l-glutamine, 1.5 g/L sodium bicarbonate, 1 mM nonessential amino acids, 50 U/mL penicillin, 50 μg/mL streptomycin, and 10% fetal bovine serum (FBS).

L5178Y *Tk*^+/−^ mouse lymphoma cells (ATCC code CRL-9518) were grown in RPMI 1640 minimal medium supplemented with 2 mM l-glutamine, 1 mM sodium pyruvate, 1 mM nonessential amino acids, 50 U/mL penicillin, 50 μg/mL streptomycin, F68 pluronic, and 10% horse serum (heat-inactivated). Cells were grown at 37 °C in a 5% carbon dioxide atmosphere (100% nominal humidity) and routinely tested for mycoplasma contamination.

Epstein–Barr immortalized lymphoblastoid cell lines LFA145^−/−^ and LFA55^−/−^ originated from patients with FA, were obtained by Prof. Jordi Surralles (Genetics Department, Hospital Sant Pau, Barcelona, Spain). The LFA145^−/−^ cell line bears the mutation c.893+920 C > A in allele 1 causing an altered splicing and a large deletion of exons 1-20 (ex1-20del) in allele 2 of *FANCA* gene with no protein produced. The LFA55^−/−^ cell line is homozygous for a point mutation (c.295C > T) in this same gene [37]. The wild-type LFA195^+/+^ lymphoblastoid cell line derived from a healthy relative of one FA patient (LFA145^−/−^) was included as a negative control. The growth kinetics of wild-type and FA lymphoblasts used in this study were similar. The lymphoblastoid cell lines were cultured in RPMI supplemented with 15% fetal bovine serum and antibiotics.

### 4.4. Treatment of Cells in Culture

Furan was dissolved in DMSO and diluted appropriately, immediately before addition to the cells. To limit as much as possible furan accumulation in the gas phase, cultures were treated with furan for 3–4 h (in serum-free medium) or 24 h (in growth medium) at 37 °C in tightly closed flasks completely filled with media. Freshly prepared solutions of BDA were immediately diluted in growth medium for cell treatment. The cells were treated for 3–4 h in serum-free medium.

### 4.5. V79 Hprt Mutation Assay

The *Hprt* mutation assay was conducted as previously reported [38]. For each treatment group, 2 × 10^6^ V79 cells were plated in 75 cm^2^ flasks the day before the experiment and allowed to attach overnight. On day 0, cells were treated for different exposure times with a wide range of furan or BDA concentrations. At the end of the incubation period, 200 cells were plated in 60 mm Petri dishes to evaluate cytotoxicity. Cells were then incubated for 6–9 days to allow the phenotypic expression. On days 6 and 9, cells (1 × 10^5^) were plated in 100 mm Petri dishes in selective medium containing 7.5 μg/mL 6-thioguanine. Cells from each culture were also plated at 200 cells per 60 mm Petri dish in triplicate in the absence of selective drug to determine plating efficiency (PE). After 2 weeks of incubation, colonies were counted and the mutation frequency was calculated [mutation frequency = number of colonies/(number of seeded cells × PE) × 10^6^ cells]. The PE of cells treated with solvent was set 100%. A 10 mM concentration of ethyl methanesulfonate (EMS) was used in each experiment as positive control. Mean mutation frequencies in control and treated cultures were compared by two-tailed Student’s *t* test.

### 4.6. Mouse Lymphoma Tk^+/−^ Mutation Assay

The mouse lymphoma *Tk*^+/−^ mutation assay (96-well version) was conducted following the method described in OECD Test Guideline N. 490 in the absence of S9 metabolism. Two replicate cultures with 1.5 × 10^5^ cells/mL (72 mL) were prepared for each test point with furan and duplicate cultures with 5 × 10^5^ cell/mL (20 mL) in the case of BDA. At the end of treatment, the cells were washed with phosphate buffer saline (PBS), a sample of 4 × 10^6^ treated cells was suspended in 20 mL of normal culture medium for a total expression period of 48 h, during which the Tk-deficient phenotype could be expressed. After 24 h, the cell density was checked, and the cell suspension was diluted again to 2 × 10^5^ cells/mL. At the end of the expression period, 1.6 cells/well were inoculated in two 96 well plates, for cloning efficiency. For mutant selection plating (density: 10,000 cells/mL), 3 μg/mL of 2-deoxy-5-trifluoromethyl-uridine was added to the suspension. Cells were dispensed at 0.2 mL/well on four 96 well plates, to distribute 2000 cells/well.

The plates were incubated for 12 days and wells containing no viable clones were identified by eye and counted. Small and large clones were recorded separately. The mutant frequency (MF) and the RTG were calculated according to [39]. Statistical significance of mutant frequencies was evaluated according to [40]. The one tailed Dunnett’s test was used to compare the mutant frequency in control and treated cultures and a linear trend analysis of mutant frequency with treatment dose was performed using weighted regression. Biological relevance of results was assessed according to criteria developed by the Mouse Lymphoma Expert Workgroup of the IWGT [41], whereby a result is considered clearly positive when the increase in mutation frequency above the concurrent background exceeds the Global Evaluation Factor (126 × 10^−6^).

### 4.7. Cytokinesis-Block Micronucleus Assay (CBMN) in the L5178Y Tk^+/−^ Mouse Lymphoma Cell Line with BDA

In order to assess the potential of BDA to induce micronuclei in L5178Y *Tk*^+/−^ mouse lymphoma cells (clastogenic or aneugenic activity), the test compound was added to cell suspensions in complete culture medium at cell density of 1.0 x 10^6^ cells/mL. Treatments were performed in the absence of S9 metabolism only with 24 h harvest time. At the end of the short (4 h) treatment time, Cytochalasin B (CytB) at final concentration of 6 µg/mL, was added and cells incubated at 37 °C for the recovery period of 21 h. In the case of 24 h continuous exposure, CytB was added to the treatment medium at start of treatment. Negative (vehicle) and positive (MMC and Vinblastine as clastogenic and aneugenic chemical, respectively) controls were included in each test. At the defined sampling time, the cells were processed following standard procedures to obtain air-dried cytogenetic preparations stained with Acridine Orange. The cytokinesis-block proliferation index (CBPI) determined from at least 500 cells per culture, was used to calculate cytotoxicity.

### 4.8. Chromosomal Aberration Assay in Fanconi Anaemia Lymphoblastoid Cell Lines

Furan and BDA were added to FA-derived lymphoblastoid cell suspensions and controls in complete culture medium at a cell density of 1 × 10^6^ cells/mL. Treatments with furan were performed both in the absence and presence of S9 metabolism. For BDA, treatments were performed only in the absence of S9 metabolism and sampling of cells was scheduled after 24 h from the beginning of treatment. A recovery period of 12 h with a sampling of cells at 36 h was also performed in order to assess DNA repair. For both furan and BDA, the dose-levels employed were selected from previous dose-range finder experiments and calculated at identical intervals between dose-levels. In the experiments with recovery, at the end of the treatments, the cells were washed twice with PBS and left to recover in culture medium for further 12 h. During the last 3 h of culture, colcemid (Gibco BRL, Crewe, Cheshire, UK) at a final concentration of 0.2 μg/mL was added to each culture. Negative (vehicle) and positive (MMC and Cyclophosphamide in the absence and presence of rat liver S-9, respectively) controls were included in each test. At the defined sampling times (24 or 36 h), cells were processed following standard procedures to obtain air-dried cytogenetic preparations and stained with Giemsa.

For each experimental point, 100 metaphases were scored in blind mode with coded slides. Chromosomal aberrations were classified according to [40]. Mitotic indices were expressed as a percentage based on the number of metaphases present after a total of 1000 cells had been scored (interphases and metaphases). For the chromosome aberration assay, the number of aberration-bearing cells (excluding gaps) was utilized for statistical analyses. To determine the statistical significance, the Fisher’s exact test was used. A test substance was considered positive when statistically significant increases in aberration-bearing cells were observed at two consecutive dose-levels or at the higher dose-level and exceeded the historical control mean values for the laboratory.

### 4.9. Animals

Male B6C3F1 mice aged 5–6 weeks were obtained from Harlan s.r.l. (Udine, Italy). *Ogg1*^−/−^ mice were a kindly provided by D. Barnes [42]. Animals were housed in a room with a barrier system under standard environmental conditions (22 ± 2 °C, 55 ± 15% relative humidity, on a 12-h light–dark cycle), with drinking water and laboratory rodent diet *ad libitum*. Experiments were carried out in compliance with the ethical provisions enforced by the European Union and authorized by the National Committee of the Italian Ministry of Health on the in vivo experimentation.

### 4.10. Treatment of Animals

For in vivo treatments fresh solutions of furan in corn oil were prepared before each experiment. Precautions were used in order to minimize evaporative losses during furan handling. Solutions of furan were quickly aliquoted in filled amber vials, one for each day of treatment, which were tightly sealed and stored at 4 °C. The actual furan concentrations of test solutions were checked by head-space capillary gas chromatography with flame ionization detector. The correlation coefficient of nominal and actual furan concentrations in corn oil solutions was >0.99.

After arrival, mice were assigned to treatment groups with similar average body weight and acclimatized 1 week in standard conditions. In repeat dose experiments, furan was administered by gavage 5 days a week for 4 weeks and animals sacrificed by cervical dislocation 24 h after last administration. The delivered volume was 10 mL/kg b.w. in all cases. Methyl methanesulphonate (MMS) (Sigma), dissolved in dimethylsulphoxide (DMSO) and administered by i.p. at 80 mg/kg b.w., was used as positive control. For the assessment of hepatocyte proliferation, satellite groups of treated and control mice were given i.p. injections of BrdU (Sigma Aldrich-Merck, Milan, Italy), 100 mg/kg bw in 0.9% NaCl, both 24 and 2 h before the sacrifice. Animals were weighted once a week along treatment period. General health conditions were recorded daily.

### 4.11. Histopathology and BrdU Incorporation Assay

After sacrifice, mouse liver specimens were excised and fixed in 4% formalin for 24 h at room temperature. Subsequently, tissue samples were embedded in paraffin, sectioned at 3 µm, stained with hematoxylin and eosin (H&E) and examined under a light microscope (DMLB light microscope; Leica Microsystems, Wetzlar, Germany).

For the assessment of BrdU incorporation, liver sections were prepared as described above, trypsinized for 30 min in a humid chamber and then heated for 10 min at 100 °C, in pH 6 citrate buffer, to induce DNA breaks. Subsequently, sections were incubated with the anti-BrdU Monoclonal Antibody (Thermo Fisher Scientific, Waltham, MA, USA, Cat# MA3-071, RRID: AB10986341) and a rabbit anti-mouse IgG secondary antibody (AP-conjugated) (Thermo Fisher Scientific Cat# A16163, RRID: AB_2534834). Lastly, sections were incubated with Fast Red Substrate System (Agilent, Santa Clara, CA, USA), for 20 min, and counterstained with haematoxylin. BrdU incorporation was assessed by light microscopy, at ×200, using the Qwin image analysis software (Leica Microsystems Srl, Milan, Italy, QWin, RRID:SCR_018940). Six to eight fields were analyzed for each section and three sections per sample were evaluated. Results were expressed as mean number ± SD of positive nuclei per field. Each slide was analyzed by two independent investigators, in a blind fashion, and there was an almost perfect agreement (kappa value = 0.80) between their evaluations [43].

### 4.12. Isolation of Splenocytes

After sacrifice, the spleen was aseptically removed and weighted. Single cell suspension was prepared through mechanical desegregation in cold RPMI 1640 medium (Gibco, Paisley, UK). Splenocytes were isolated by stratification on Hystopaque 1077 (Sigma Aldrich-Merck, Milan, Italy), followed by centrifugation for 30 min at 390× *g*. After isolation, cells were washed twice in RPMI 1640 by centrifugation (10 min at 200× *g*) and used to set up duplicate cultures for micronucleus.

### 4.13. Cytokinesis-Block Micronucleus Assay (CBMN) In Vivo

Splenocytes isolated as described above were used to set up cell cultures with 2 × 10^6^ cells/mL in RPMI 1640 medium with Hepes and Glutamine (Gibco, Paisley, UK) supplemented with 1% streptomycin-penicillin, 15% inactivated calf serum and Concanavalin A (Sigma Aldrich-Merck, Milan, Italy) at a final concentration of 5 μg/mL. CytB (Sigma) was added 24 h after culture onset at a final concentration of 5 μg/mL. Binucleated cells were harvested after further 10 h of culture using a cytocentrifuge (Shandon, Waltham, MA, USA). After fixation and Giemsa staining, slides were coded and blindly scored using a brightfield microscope. The frequency of micronucleated binucleated (MnBn) splenocytes was evaluated in 1000 binucleated cells/animal. To evaluate the rate of in vitro proliferation of stimulated spleen lymphocytes the NDI (binucleated + 2 × multinucleated cells/total analyzed cells) was determined. Mean frequencies of micronucleated splenocyte in different experimental groups were compared by two-tailed Student’s *t* test. The limit for statistical significance was set at *p* = 0.05.

### 4.14. Preparation of Liver, Spleen, Kidney, Brain, Small Intestine, and Lung DNA

Mice were killed by cervical dislocation, and excised organs were washed with ice-cold PBS. Liver was diced and washed with hypotonic KCl. Washed tissues were snap-frozen in liquid nitrogen. Before DNA extraction, thawed tissues were finely minced in lysis buffer [10 mm Tris HCl (pH 8.0), 10 mm EDTA, 10 mm NaCl, and 0.5% SDS]. DNA was extracted by a high-salt protein precipitation method. Briefly, following lysis tissues were digested with RNase at 37 °C for 1 h and protease (Qiagen Srl, Milan, Italy) at 37 °C overnight. Proteins were precipitated by adding NaCl to 1.5 m, and DNA in the supernatant was collected by addition of 2 vol of ethanol.

### 4.15. Measurements of DNA 8-oxodG

8-OxodG was measured by high-performance liquid chromatography with electrochemical detection (HPLC/EC) as described previously [33]. Briefly, DNA was resuspended in Tris-EDTA, incubated with RNases A and T1 at 37 °C for 1 h, and precipitated again with ethanol. Enzymatic digestion was then performed at 37 °C using nuclease P1 (Boehringer Mannheim) for 2 h and alkaline phosphatase (Boehringer Mannheim, Mannheim, Germany) for 1 h. Enzymes were precipitated by addition of CHCl3, and the upper liquid layer stored at −80 °C under N2 for subsequent analysis 8-oxodG. The DNA hydrolysate was analyzed by high-performance liquid chromatography with electrochemical detection (Coulochem I; ESA, Inc., Chelmsford, MA, USA) using a C18 250 × 46 mm 5-μm Uptishere column (Interchim) equipped with a C18 guard column. The eluent was 50 mm ammonium acetate (pH 5.5), containing 9% methanol, at a flow rate of 0.7 mL/min. The potentials applied were 150 and 400 mV for E1 and E2, respectively. The retention time of 8-oxodG was ~23 min. Deoxyguanosine was measured in the same run of corresponding 8-oxodG with a UV detector (model SPD-2A; Shimadzu, Kyoto, Japan) at 256 nm; the retention time was ~17 min.

## Figures and Tables

**Table 1:** *Tk*^+^/*Tk*^−^ mutations induced by 24 h exposure to furan in L5178Y cells.

Dose (mM)	RTG (%)	MF (×10^−6^ Cells)	Induced MF (×10^−6^ Cells)	Small Colonies MF (×10^−6^ Cells)	Large Colonies MF (×10^−6^ Cells)	Small/Large Colonies (%)
0	100	90.7	-	38.4	48.9	0.44
1.78	109	70.6	0	32.0	41.1	0.44
2.37	133	74.2	0	28.3	40.7	0.41
3.13	125	61.7	0	15.2	40.8	0.27
4.22	97	72.3	0	30.5	38.6	0.44
5.62	96	76.1	0	29.2	42.9	0.41
7.50 ^§^	3	294.5	203.8	182.3	94.7	0.66
Linear trend		NS		NS	NS	
MMS 45.4 × 10^−3^	125	344.1 **	253.4	154.5 **	131.3 **	0.54
0	100	66.8	-	14.4	50.6	0.22
3.06	82	55.6	0	15.4	38.7	0.28
3.35	70	74.5	7.71	16.1	56.0	0.22
3.67	51	75.0	8.24	18.4	54.7	0.25
4.03	46	80.4	13.60	28.4	49.5	0.36
4.42	50	103.4	36.62	27.5	72.3	0.28
4.84	24	113.2	46.43	32.2	77.5	0.29
5.31 ^§^	11	145.0	78.16	65.1	71.2	0.48
5.82 ^§^	8	127.5	60.68	51.4	70.0	0.42
Linear trend		* *p* < 0.05		* *p* < 0.05	NS	
MMS 45.4 × 10^−3^	125	424.4 **	357.6	119.2 **	242.4 **	0.54

^§^ Treatment excluded from statistical analysis due to extreme cytotoxicity; * *p* < 0.05; ** *p* < 0.01; RTG, Relative Total Growth; MF, Mutation Frequency; MMS, Methyl methanesulfonate.

**Table 2:** *Tk*^+^/*Tk*^−^ mutations induced by 24 h exposure to BDA in L5178Y cells.

Dose (μM)	RTG (%)	MF (×10^−6^ Cells)	Induced MF (×10^−6^ Cells)	Small Colonies MF (×10^−6^ Cells)	Large Colonies MF (×10^−6^ Cells)	Small/Large Colonies (%)
0	100	57.9	-	21.7	35.3	0.38
7.5	84	68.6	10.71	32.0	34.5	0.48
10	85	70.4	12.50	21.4	46.5	0.32
15	93	71.3	13.38	25.6	42.9	0.37
20	82	82.8	24.95	28.9	50.4	0.36
40	64	118.7 **	60.86	64.9 **	45.5	0.59
80 ^§^	6	268.4	210.5	133.8	109.3	0.55
Linear trend		***		***	NS	
MMS 90.8	88	343.1 **	285.2	136.6 **	155.7 **	0.47
0	100	62.0	-	26.3	34.3	0.43
20	110	63.9	1.86	29.5	33.3	0.47
30	105	66.5	4.53	27.9	37.6	0.43
40	65	87.0	25.01	49.6	36.4	0.58
50 ^§^	10	483.2	421.2	248.1	200.9	0.55
60 ^§^	5	516.5	454.5	302.4	182.3	0.62
Linear trend		NS		NS	NS	
MMS 90.8	127	244.3 **	182.3	117.3 **	79.1 *	0.60

^§^ Treatment excluded from statistical analysis due to extreme cytotoxicity; * *p* < 0.05; ** *p* < 0.01; *** *p* < 0.001; NS, not significant; RTG, Relative Total Growth; MF, Mutation Frequency; MMS, Methyl methanesulfonate.

**Table 3:** Micronuclei induced by BDA in L5178Y cells.

Treatment	Dose (µM)	Cytotoxicity (%)	MnBn Cells (‰)	Total Micronuclei	Distribution of Micronuclei			
					0	1	2	3
4 h treatment								
Untreated	0	-	8	16	1984	16	-	-
BDA	10	10	14	28	1972	28	-	-
BDA	15	23	22 ***	44	1956	44	-	-
BDA	20	27	18 *	36	1964	36	-	-
24 h treatment								
Untreated	0	-	12	24	1976	24	-	-
BDA	10	49	20	40	1960	40	-	-
BDA	15	55	27 ***	54	1947	52	1	-
BDA	20	62	36 ***	75	1931	69	3	-
MMC	1.5 μg/mL	2	24 ***	51	1952	46	1	1

Cytotoxicity = 100–100 [(CBPI_T_-1)/(CBPI_C_-1)]; CBPI_T_ = Cytokinesis-block proliferation index in treated cultures; CBPI_C_ = Cytokinesis-block proliferation index in negative control cultures. MnBn, Micronucleated binucleated cells; * *p* < 0.05; *** *p* < 0.001; MMC, Mitomycin C.

**Table 4:** Body weight gains and organ weights of C57Bl6 mice after 4 weeks of furan treatment.

Furan (mg/kg bw)	Survived Mice (No.)	Final Body Weight (g)	Liver		Spleen	
		Mean ± SE	Weight (g) Mean ± SE	Hepatic ^a^ Index Mean ± SE	Weight (mg) Mean ± SE	Splenic ^a^ Index Mean ± SE
0	5	23.3 ± 1.4	1.03 ± 0.1	4.40 ± 0.6	73.9 ± 11.5	0.32 ± 0.5
8	6 ^§^	24.1 ± 0.4	1.29 ± 0.1	5.37 ± 0.3 *	84.2 ± 8.6	0.35 ± 0.02
15	5	22.6 ± 2.7	1.25 ± 1.2	5.50 ± 0.4 **	85.1 ± 24.4	0.39 ± 0.15
30	6	22.0 ± 0.9	1.18 ± 0.3	5.31 ± 1.3	75.7 ± 16.4	0.34 ± 0.08

* *p* < 0.05; ** *p* < 0.01 (Student *t* test); ^§^ One mouse from this group died during treatment; ^a^ The organ index formula is: organ weight (g)/body weight (g) × 100.

**Table 5:** Cytogenetic analysis of cytokinesis-blocked splenocytes of C57Bl6 mice following 4-week exposure to furan.

			MnBn	
	Furan Dose	Mice	MnBn/1000 Cells	Frequency
	mg/kg bw	No.	Individual Animal Data	Group Mean ± SE
Exp. 1	0	5	5, 3, 2, 5, 1	3.2 ± 0.8
	8	5	6, 1, 9, 8, 8	6.4 ± 1.4
	15	5	8, 9, 6, 3, 7	6.6 * ± 1.1
	30	6	7, 16, 7,10, 6, 9	9.2 ** ± 1.5
Exp. 2	0	6	2, 4, 2, 1, 3, 2	4.6 ± 0.8
	8	6	1, 3, 5, 3, 7, 4	7.6 ± 0.4
	15	6	6, 3, 3,4, 7, 3	8.6 * ± 1.1
	30	5	12, 5, 10, 8, 9	17.6 ** ± 1.5

* *p* < 0.05; ** *p* < 0.001 (Student *t* test); MnBn, Micronucleated binucleated cells.

## Data Availability

Not applicable.
